# Content-Based Estimation of Brain MRI Tilt in Three Orthogonal Directions

**DOI:** 10.1007/s10278-020-00400-7

**Published:** 2021-02-24

**Authors:** Pooja Prabhu, A. K. Karunakar, Sanjib Sinha, N. Mariyappa, G. K. Bhargava, J. Velmurugan, H. Anitha

**Affiliations:** 1 Department of Computer Applications, Manipal Institute of Technology, Manipal Academy of Higher Education, Manipal, Karnataka 576104 India; 2 Department of Neurology, National Institute of Mental Health and Neurosciences, Bengaluru, Karnataka 560029 India; 3 MEG Research Centre, National Institute of Mental Health and Neurosciences, Bengaluru, Karnataka 560029 India; 4 Department of Clinical Neurosciences, National Institute of Mental Health and Neurosciences, Bengaluru, Karnataka 560029 India; 5 Department of Electronics and Communication Engineering, Manipal Institute of Technology, Manipal Academy of Higher Education, Manipal, Karnataka 576104 India

**Keywords:** Magnetic Resonance Imaging (MRI), Principal Component Analysis (PCA), Multimodality Registration, Rotational Effect

## Abstract

In a general scenario, the brain images acquired from magnetic resonance imaging (MRI) may experience tilt, distorting brain MR images. The tilt experienced by the brain MR images may result in misalignment during image registration for medical applications. Manually correcting (or estimating) the tilt on a large scale is time-consuming, expensive, and needs brain anatomy expertise. Thus, there is a need for an automatic way of performing tilt correction in three orthogonal directions (*X*, *Y*, *Z*). The proposed work aims to correct the tilt automatically by measuring the pitch angle, yaw angle, and roll angle in *X*-axis, *Z*-axis, and *Y*-axis, respectively. For correction of the tilt around the *Z*-axis (pointing to the superior direction), image processing techniques, principal component analysis, and similarity measures are used. Also, for correction of the tilt around the *X*-axis (pointing to the right direction), morphological operations, and tilt correction around the *Y*-axis (pointing to the anterior direction), orthogonal regression is used. The proposed approach was applied to adjust the tilt observed in the T1- and T2-weighted MR images. The simulation study with the proposed algorithm yielded an error of 0.40 ± 0.09°, and it outperformed the other existing studies. The tilt angle (in degrees) obtained is ranged from 6.2 ± 3.94, 2.35 ± 2.61, and 5 ± 4.36 in *X*-, *Z*-, and *Y*-directions, respectively, by using the proposed algorithm. The proposed work corrects the tilt more accurately and robustly when compared with existing studies.

## Introduction 

The brain is a complex organ with several distinct anatomical structures. The brain’s pathological conditions that are anatomically appealing are epilepsy, Alzheimer’s disease, stroke, tumor, hemorrhage, etc. The clinical diagnosis of these pathological conditions involves imaging modalities like magnetic resonance imaging (MRI), computed tomography (CT), and positron emission tomography (PET). The brain images are tilted [1] when acquired through these modalities rather than aligned with the scanner. Visual inspection of such images by clinicians while diagnosis can lead to erroneous perception of abnormalities [2–4]. The tilt in the brain images could be due to the movement of the subject’s head inside the scanner, improper data calibration, and inexperience by the operator.

In a few studies [5, 6], for a better understanding of the location of the abnormalities within the brain tissue, multimodality registrations [7–10] are performed. In multimodality registration, images from different modalities are brought into a common coordinate frame [11]. The brain images used for registration must be undistorted; in other words, the images need to be accurately aligned [5] within the scanners’ coordinate system. In most of the cases, the images acquired are not aligned but are tilted. The tilt experienced by the images is not easily detectable because of not knowing the actual reason for the tilt. When dealing with medical images on a large scale, manual tilt correction becomes expensive and time-consuming. Also, the labor performing tilt correction should have an adequate amount of knowledge on brain anatomy. Thus, there is a need for a computer-aided algorithm to correct the tilt.

Generally, four approaches exist for tilt corrections: shape-based, content-based, 2D-based, and 3D- based [12]. Since the tilt correction is essential for various clinical applications, several studies exist related to this area [13]. Shape-based methods use geometrical landmarks like cerebral Inter-hemispheric Fissure (IF) [14] to estimate the head’s alignment. Content-based methods explore the brain’s bilateral symmetry by matching the intensities of one half of the brain with the other half of the brain. The features to match either side of the brain can be intensity values [15] or edge images [16]. In 2D-based methods, extracted 2D lines from each slice helped in the computation of a 3D plane using the interpolation techniques [17]. In 3D-based methods, the tilt correction is performed by the plane that maximizes the bilateral symmetry by considering the head as a whole volume [18]. The proposed study employed the content-based techniques to correct the tilt in *X*, *Y*, *Z*-directions of brain MR images.

## Related Works

Various works exist in the estimation of tilt angles using content-based methods. Ardekani et al. [18] evaluated the tilt angle performing a cross-correlation between the image and its reflected version. They considered the image, rotated in the range − 10 to 10°, and the angle at which the image gives the largest cross-correlation value is the tilt observed in *Y*-direction and *Z*-direction. This technique fails to provide the correct tilt angle if the image shows asymmetry due to the pathological effects. Liu et al. [19] used edge detection on axial slice followed by cross-correlation to estimate Z-direction tilt. The study by Liu et al. [19] computed the tilt in *Y*-direction by fitting the plane perpendicular to the set of an axial slice. Tilt correction failed when the head rotates beyond 20°. Prima et al. [20] performed the block matching technique on the two hemispheres of head volume. The block, which shows the maximum similarity, exhibits the symmetry of the brain. This study used the intensity-based techniques to match intensity between each block, which is time-consuming. Besides, the study does not report the correct tilt when pathological conditions exist. Ruppert et al. [21] used a 3D Sobel edge detector followed by correlation for either side of the hemispheres. The estimation of the tilt angle in *Y*-direction and *Z*-direction uses coronal slice and axial slice, respectively. This technique is sensitive to noise and did not report tilt correction under pathological conditions. Wu et al. [22] used a 3D scale-invariant feature transform followed by correlation to estimate the tilt in *Y*-direction and *Z*-direction. This method is sensitive to noise, asymmetry, and blur. Kalavathi et al. [23] used skull stripped brain images, followed by curve fitting. Even though it is robust and straightforward compared with previous works, it fails to correct the tilt if the rotation angle exceeds 15°. Rehman et al. [24] estimated the tilt in *Y*-direction and *Z*-direction using principal component analysis (PCA) and cross-correlation on T1-weighted brain MR images. Further, their algorithm requires a manual intervention to select axial slices from the middle few slices, and they did not consider the T2-weighted brain MR images. Previous works estimated tilt in *Y*-direction and *Z*-direction by assuming that the subject inside the MRI aligned with respect to the MR scanner’s major axis. In other words, the previous study assumes that there is no rotation about *X*-direction.

The objectives of the present study are (i) to estimate tilt angle in all the three directions and correcting tilt to obtain the aligned MR images; (ii) comparison of the proposed algorithm, on the simulated dataset, with the previous two works by Ruppert et al. [21] and Rehman et al. [24]; and (iii) assessing the robustness of the proposed approach on T1-weighted and T2-weighted MR images that has pathological involvement of lesion.

## Materials and Methods

Simulation study includes the standard dataset, with tilt free images from the Internet Brain Segmentation Repository (IBSR) [25]. These datasets comprised eighteen subjects T1-weighted images from all age groups and consisted of 128 coronal slices each of 256 × 256 dimensions (slice thickness = 1.5 mm). Besides, our algorithm considered T1-weighted images and T2-weighted images from BrainWeb Simulated Brain Database [26], which are tilt free consists of the T1-weighted image each of normal cases and pathological case (Multiple sclerosis), and one T2-weighted image of the normal. All the MR images were of dimension 217 × 181, the number of slices = 181.

Further, this study has included fourteen epileptic patients (age range from 8 to 42 years) brain MRI acquired in part of the magnetoencephalography (MEG) protocol at National Institute of Mental Health and Neurosciences (NIMHANS). These patients underwent a T1-weighted three-dimensional (3D) magnetization-prepared rapid acquisition gradient-echo sequence (repetition time = 650 ms, echo time = 14 ms, slice thickness = 1.0 mm) in a 3 T MRI machine with Vitamin-E marker over the fiducials. It consisted of 192 sagittal slices, and each slice is of dimension 256 × 256. These sagittal slices were transformed to other views, forming 256 axial slices and coronal slices, each of 256 × 192 dimensions. Institute Ethical Committee at NIMHANS approved this study.

### Tilt Correction in Brain MR Images

**Fig. 1:**
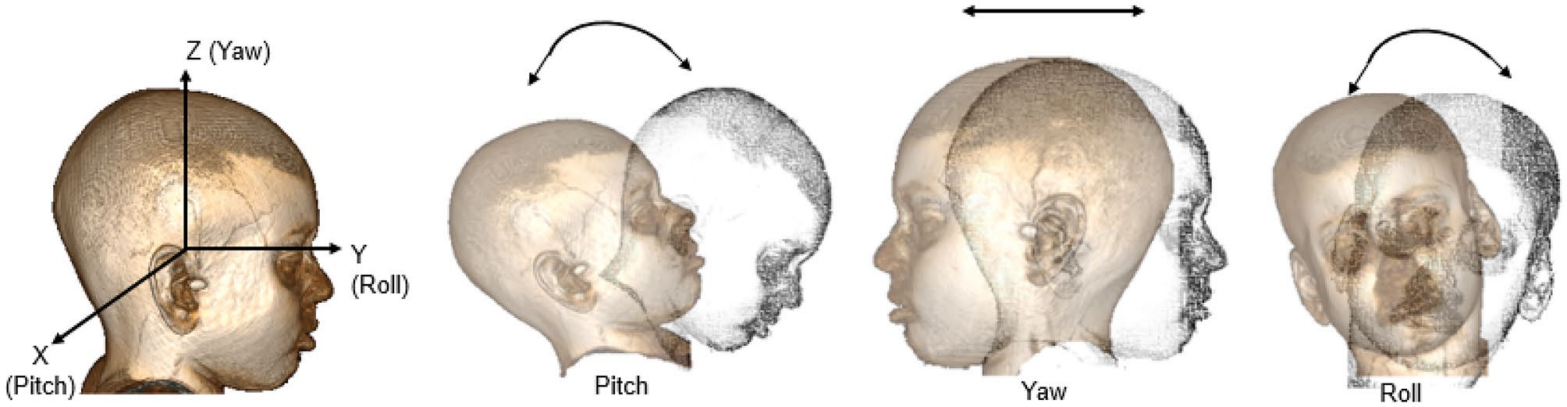
Head coordinate system demonstrating pitch angle, yaw angle, and roll angle

Generally, brain MR images’ 3-D volumetric data consists of three orientations: axial, coronal, and sagittal. The imaging coordinate system (*X*, *Z*, *Y*) varies from the ideal coordinate system because of rotation. Figure 1 depicts the rotations in three orthogonal directions in the head coordinate system. The pitch angle, yaw angle, and roll angle are the angle of rotation observed about *X*- (pointing to the right), *Z*- (pointing to superior), and *Y*- (pointing to anterior), respectively. The angle of rotation obtained for each direction is used to correct the tilt using the rotation matrix. The order in which the rotation angle estimated does not matter because we used the original MR volume to estimate each rotation angle.

### Pitch Angle (*ω*)

**Fig. 2:**
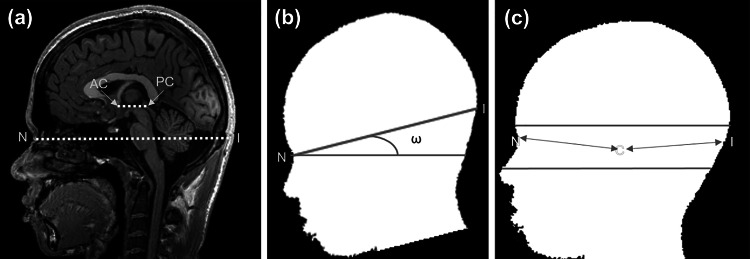
Estimating the pitch angle: **(a)** relationship between the AC-PC line and line joining the Nasion (N) and Inion (I) point, **(b)** estimation of pitch angle (*ω*) with respect to the line joining the Nasion (N) and Inion (I), and **(c)** in anterior, Nasion (N) is the point that has a minimum distance from Centroid (C), and in posterior, Inion (I) is the point that has the maximum distance from the Centroid (C) (the Nasion and Inion point marked by visual inspection)

The brain aligned in *X*-direction will have a line joining the Anterior Commissure (AC) to Posterior Commissure (PC) parallel to *Y*-axis [27]. When the head is correctly aligned, the line joining from the Nasion (N) to the Inion (I) will be parallel to the line joining of AC and PC (as shown in Fig. 2a). The angle made by the line joining the Nasion to the Inion is the tilt observed about *X*-direction. This study uses the idea as mentioned above to calculate the angle of rotation observed about *X*-direction. For example, if the head suffers the tilt about *X*-direction (as shown in Fig. 2b), then the line joining the Nasion to Inion also suffers the same tilt. The angle formed by the line joining Nasion to Inion with *Y*-axis gives the angle of rotation about *X*-direction.

**Fig. 3:**
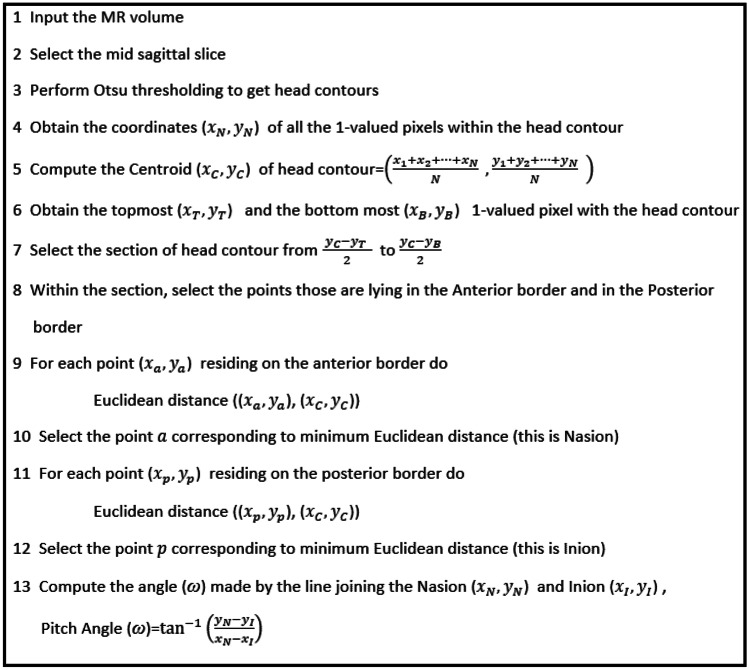
Pseudo-code proposed to estimate the pitch angle

The pitch angle is the angle of rotation observed about *X*-direction. Evaluation of this angle considers the mid-sagittal slice that shows the midbrain structures (like medulla oblongata and pons) and corpus callosum with AC and PC. Dividing the total number of sagittal slices of 3D-volumetric brain MRI data by 2 can obtain a mid-sagittal slice. The histogram of MR images showed the bimodal distribution due to which mid-sagittal slice is subjected to Otsu thresholding [28, 29] to get the head contour. The head contour’s centroid is computed based on the number of pixels (of value 1) within the head contours. There are three points in the head contour’s anterior part with a minimum distance from the Centroid; they are Nasion, Philtrum, and labial Commissure. A percentage of pixels above and below the Centroid is obtained that encloses only Nasion. After several observations, we chose half of the section between the top of the contour and the Centroid, and from the Centroid to the half of the section between the Centroid and bottom of the contour. Suppose we choose the section beyond this limit; in that case, the section will include Philtrum and labial Commissure, which increases the ambiguity in choosing the correct Nasion point because Philtrum and labial Commissure also have a minimum distance from the Centroid. The range is considered based on the assumption that the Nasion point and Inion point lies within this range. Then, we computed Euclidean distance between the Centroid and the set of points located at the head contour’s anterior boundary within this range. The Nasion (N) point is the one that has a minimum distance from Centroid (C) (as shown in Fig. 2c). Compute Euclidean distance between the Centroid and the set of points located at the head contour’s posterior boundary within this range. Anatomically, the Inion point is the crest present at the posterior part of the skull [30], so the Inion point is the one that has a maximum distance from the Centroid (C). Then, compute pitch angle (*ω*) by finding the angle made by the line joining the Nasion point and Inion point with horizontal (or *Y*-axis). The line joined the Nasion, and Inion varied by 15 mm ± 2.3 mm. The Nasion and Inion point identified by the proposed algorithm varied by 1 mm ± 0.5 mm and 3 mm ± 0.75 mm. Visual inspection of Nasion is easier than Inion because of the major depression felt between the eyes. To estimate the pitch angle using the proposed algorithm, one must ensure that MR volume should have a Nasion point. Figure 3 illustrates the pseudo-code for estimating the pitch angle.

### Yaw Angle (*θ*)

Yaw angle is the angle of rotation observed about *Z*-direction. This study uses the axial slices to estimate the yaw angle because, in the axial slice, one can witness the nearly elliptical shape of the human brain. PCA [31] was used on these slices to align the slice in the direction in which one can observe maximum variance. Thus, PCA gives the coarse value of the yaw angle.

Unlike the previous studies [18–24], there is no manual intervention involved in this study to choose the axial slice that exhibits the elliptical shape. Instead, this study automatically chooses the axial slices that show the elliptical shape [32] using the ellipse’s properties. In this study, we considered the whole set of axial slices to estimate the yaw angle. However, inside the MR scanner during the scanning, the subject moving head will result in each slice having a different angle of rotation about *Z*-direction (yaw angle). Thus, we calculated the yaw angle on the set of axial slices with the shape of an ellipse. The final yaw angle is the average angle obtained for each axial slice.

**Fig. 4:**
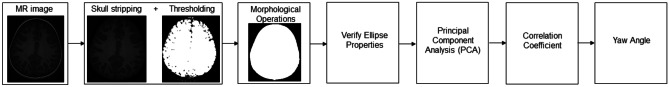
Workflow depicts the estimation of the yaw angle

By performing the workflow on the axial slices of 3D volumetric data, as shown in Fig. 4, we calculated the yaw angle. The axial slice was subjected to the skull stripping to detach the brain from the background. Otsu’s thresholding method [28, 29] binarizes the skull stripped slices. The noise in the slice was removed by performing a morphological opening. We empirically determine the structural element for morphological opening as flat, which is of size 3. By detecting the rectangular bounding box around the morphologically opened image, we extracted brain contour. By finding the first and last nonzero values along the column and the row of the morphologically opened image (*I*_1_), we mark the rectangular bounding box. Using pixel-wise multiplication of *I*_1_ and rectangular bounding box, we remove similarly valued pixels between *I*_1_ and the rectangular bounding box. The flood-fill operation filled the holes in the resulting image, which resulted in a brain contour with all the pixels of value equal to 1. The resulting image consists of the brain contour. Since the brain needs to be nearly elliptical shape to calculate the yaw angle using PCA, the resulting brain contour needs to satisfy the ellipse’s properties. The following properties of the ellipse were considered: (i) The ratio of the semi-major axis to the semi-minor axis is greater than 1.2, (ii) the semi-major axis is not equal to the semi-minor axis, and (iii) the calculated area of the ellipse (using semi-major axis and semi-minor axis) is approximately equal to the actual area of the ellipse. By multiplying the number of pixels of value equal to 1 present within the head contour with each pixel’s dimension, we obtain the actual area of the ellipse. Empirically, the resulting head contour is exhibiting an elliptical shape whose theoretical area differs within ± 200 units with that of the actual area. Beyond this range, the brain contour is not elliptical shape.

**Fig. 5:**
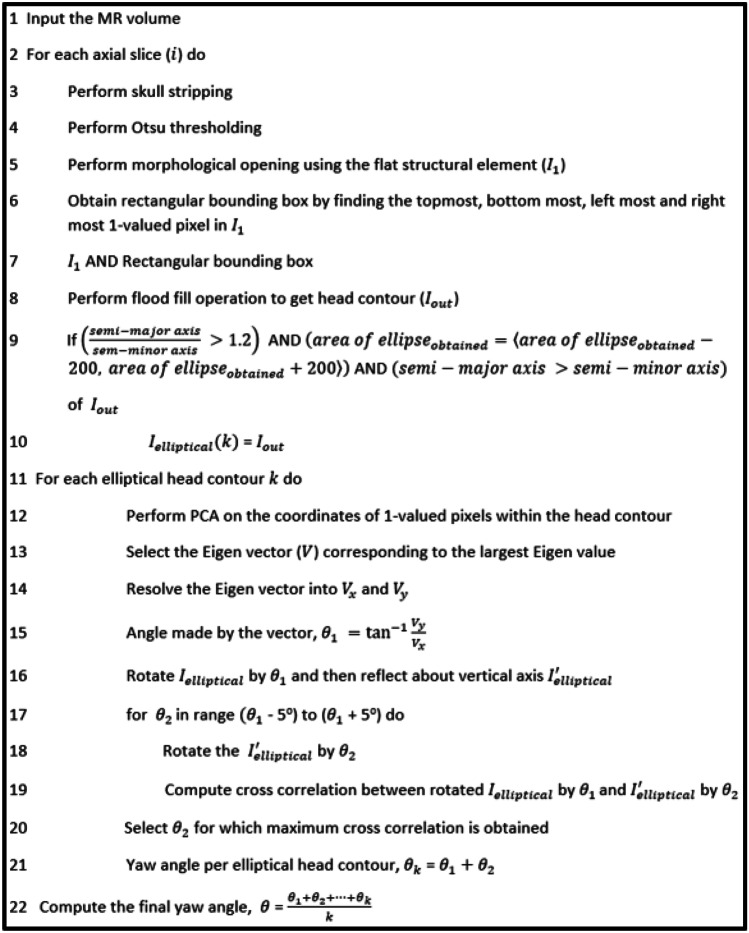
Pseudo-code proposed to estimate the yaw angle

We calculated the yaw angle on those brain contours that satisfy all three conditions. For each brain contour, The yaw angle is estimated in two steps [24], the coarse value of the yaw angle is estimated from PCA, followed by the fine value of the yaw angle estimated from similarity-based measures (like Karl Pearson correlation coefficient [33]). PCA was performed on the brain contour exhibiting the elliptical shape. PCA results in two eigenvalues associated with two eigenvectors. The two eigenvalues correspond to the semi-major and semi-minor axis of the brain contour. The angle between the eigenvector’s resolved components corresponding to the largest eigenvalue gives the approximate yaw angle (in other words, the angle made by the semi-major axis). Then, we performed a correlation on the original axial slice and its reflected version for an interval of 0.5° in the range of − 5 to 5° [24]. We obtain the maximum correlation coefficient for a given angle when we achieve the maximum bilateral symmetry on either side of the vertical axis of the image. For attaining the yaw angle for each brain contour, we sum up the angle obtained from the maximum correlation coefficient and angle of rotation obtained from PCA. The MR volume’s final yaw angle was obtained by averaging the yaw angle obtained for each brain contour. Figure 5 illustrates the pseudo-code for estimating the yaw angle.

### Roll Angle (*ϕ*)

Using orthogonal regression [34] on the set of mid parallel lines extracted from the middle few elliptical slices (in axial view), we calculated the roll angle based on the work by Rehman et al*.* [24]. In the proposed work, based on those axial slices that satisfy the ellipse’s properties, we calculate the roll angle. Then, we compute the first principal component of each slice using the PCA. The first principal component is the one that shows the highest variance. Then, we computed the top, center, and bottom coordinates for each slice where this principal component intersects. The top and bottom point corresponds to the first point of the slice with nonzero pixels along the rows, and the center point corresponds to the midpoint of these two lines. All these coordinates were subjected to orthogonal regression [34] to fit the plane to each slice’s principal component. The tilt suffered by the fitted plane is the tilt suffered in *Y*-direction. Thus, calculate the roll angle by obtaining the normal vector to two principal components of each slice. Orthogonal regression results into three column vectors, the first two are the two principal components, and the last column will be the normal vector of the two principal components. Normal vector has three coefficients, the last coefficient (say *c*) will be used to estimate the roll angle (*ϕ*) (for a detailed description refer to [19, 24, 34]) by

1 $$\phi = \mathrm{tan}^{-1} (\mathrm{c})$$

We perform tilt correction by rotating the brain MR volume in all the three directions, by substituting yaw (*θ*), pitch (*ω*), and roll (*ϕ*) angle in the rotational matrix *R* [19, 24] as follows,

2 $$R=\left[\begin{array}{ccc}\mathrm{cos}\varphi \mathrm{cos}\theta & \mathrm{cos}\theta \mathrm{sin}\omega \mathrm{sin}\varphi -\mathrm{cos}\omega \mathrm{sin}\theta & \mathrm{cos}\omega \mathrm{cos}\theta \mathrm{sin}\varphi +\mathrm{sin}\omega \mathrm{sin}\theta \\ \mathrm{sin}\theta \mathrm{cos}\varphi & \mathrm{cos}\omega \mathrm{cos}\theta +\mathrm{sin}\theta \mathrm{sin}\omega \mathrm{sin}\theta & \mathrm{cos}\omega \mathrm{sin}\theta \mathrm{sin}\varphi -\mathrm{sin}\omega \mathrm{cos}\theta \\ -\mathrm{sin}\varphi & \mathrm{sin}\omega \mathrm{cos}\varphi & \mathrm{cos}\omega \mathrm{cos}\varphi \end{array}\right]$$

Using affine transformation [35], obtain the rotation matrix. By multiplying brain MR volume with the rotation matrix (*R*), we align the tilted MR volume. On rotating the MR volume in three directions, the head volume may spread outside the image space. In order to reshape the MR volume, interpolation is performed in all three directions using trilinear interpolation.

## Results and Discussion

Our study considered three types of MR volumes viz. (i) an IBSR dataset that suffers no tilt [25], (ii) a Brainweb Simulated Brain Database [26] that suffers no tilt, and (iii) a NIMHANS dataset that suffers tilt.

### Evaluation of IBSR Database

**Fig. 6:**
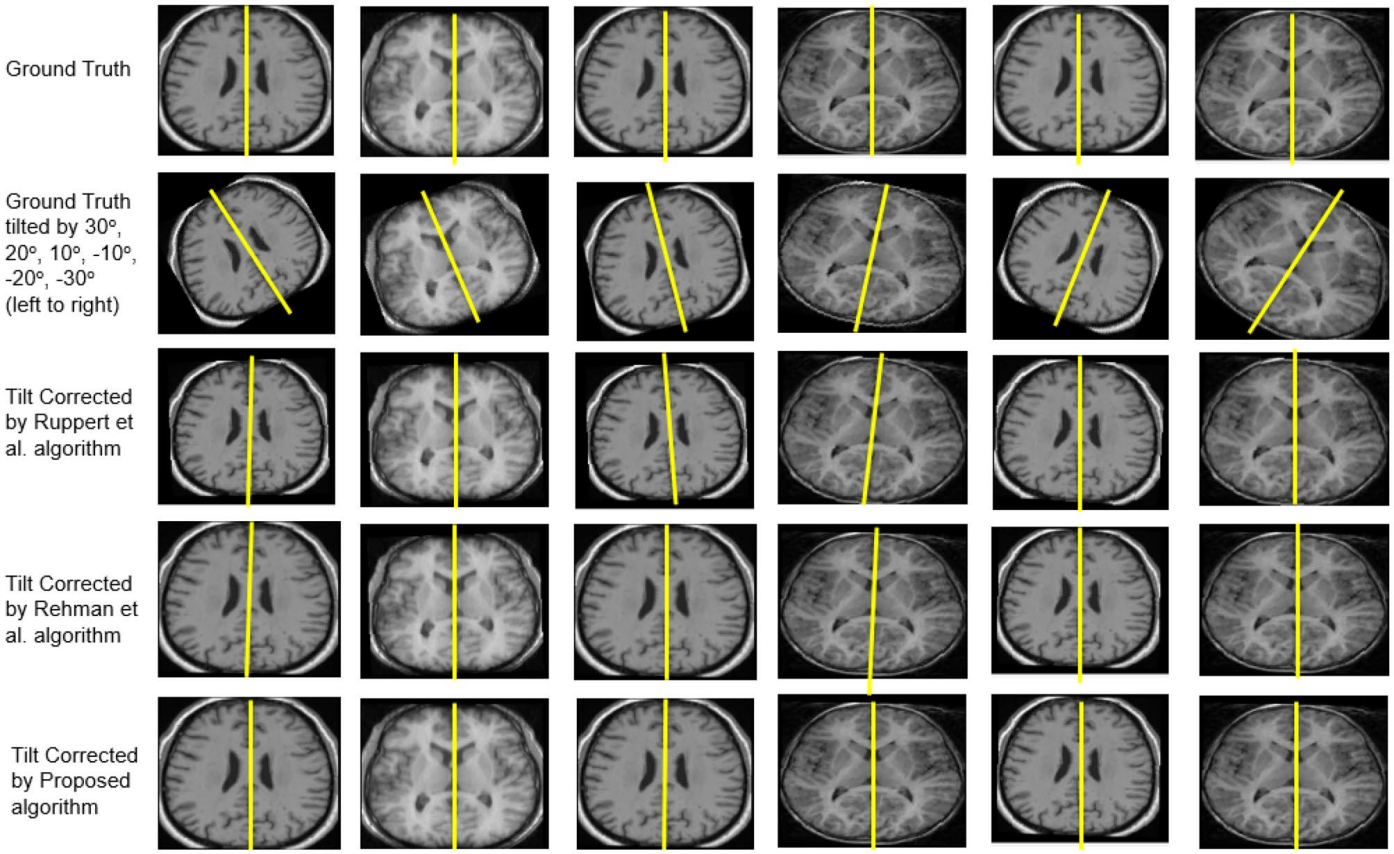
Comparison of tilt correction performed on the IBSR dataset using the proposed algorithm, Ruppert et al.[21] algorithm, and Rehman et al.[24] algorithm

We considered the MR images from the IBSR database [25] as ground truth, and also, we compared the two existing algorithms by Ruppert et al. [21] and Rehman et al. [24] with the proposed algorithm. Figure 6 shows the result obtained after correcting the tilt using existing algorithms and the proposed algorithm on the IBSR dataset.

Tilt the ground truth images by the angle of 30°, 20°, 10°, − 10°, − 20°, and − 30° in the *Z*-direction and *Y*-direction, and then these tilted images were used to correct the tilt using the algorithm by Ruppert et al. [21], Rehman et al*.* [24] and the proposed algorithm. The tilt angle’s overall variation is the average tilt angle obtained for *Z*-direction and *Y*-direction for each algorithm. During the MRI session, the subject undergoing MRI can rotate the head for an angle less than 30° because the subject’s head is attached to the MRI head frame. Thus, this study verified the proposed algorithm for (30°, − 30°) tilt in *Z*-direction.

**Fig. 7:**
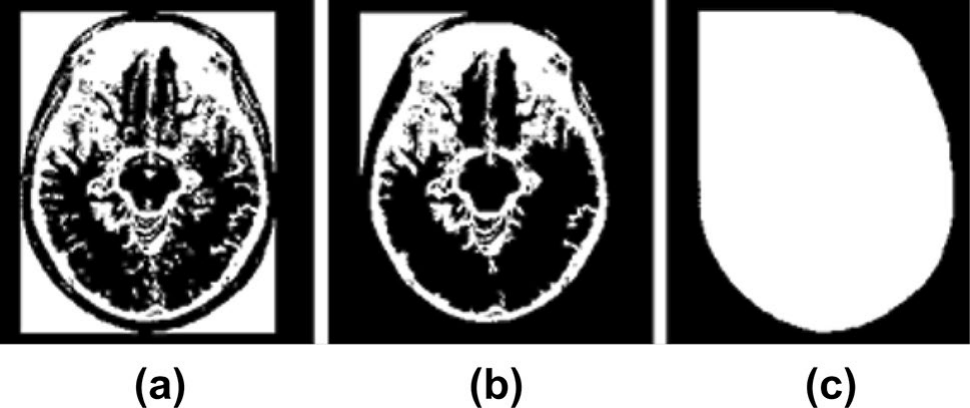
Extraction of Region of Interest (ROI): (**a**) Multiplication of image resulted from the morphological opening with the rectangular bounding box, (**b**) largest connected component, and (**c**) image obtained after flood-fill operation

When Ruppert et al. [21] algorithm was applied to correct the tilt in eighteen MR volumes, the algorithm successfully corrected tilt in *Z*-direction and *Y*-direction for fifteen subjects for the tilt angle of 20°, 10°, − 10°, and − 20°, and thirteen subjects for the tilt angle 30°, − 30°. The reason for misalignment is the blurriness of the image, which could not detect the edges precisely. The overall variation in the tilt angle after the correction was 1.2° ± 0.53°, while the algorithm by Rehman et al. [24] successfully corrected the tilt of sixteen subjects for all the tilt angles. The algorithm failed to align the two MR volumes because of the good resolution of the images, failing to extract the elliptical shape of the brain contour (as shown in Fig. 7). In good contrast MR images, for non-skull stripped images, resulting after performing the largest connected component, will have a bounding box along with the brain contour (as shown in Fig. 7c). In other words, the shape of the brain contour gets distorted. The cause for distortion is that post morphological operation results in the skull getting connected to the rectangular bounding box instead of the brain (Fig. 7b). To break the connection of the skull with brain tissues, we perform skull stripping before morphological operations. The tilt angle’s overall variation is 1° ± 0.3° while using the method employed by Rehman et al. [24]. We tested the proposed algorithm on eighteen MRI volumes obtained from the IBSR database, out of which seventeen MR volumes resulted in the same angle (30°, 20°, 10°, − 10°, − 20°, − 30°) as simulated. The overall variation of tilt obtained on using the proposed algorithm is 0.43° ± 0.09°. Thus, the proposed approach is accurate, and the error in correcting the tilt does not exceed more than a degree.

Further, we tested the algorithms by Ruppert et al. [21] and Rehman et al. [24] and the proposed algorithm on T1-weighted MR images corrupted with the variation of 1%, 3%, 5%, 6%, 7%, 8%, and 9% of noise. All three algorithms were unsuccessful in correcting the tilt when the noise level beyond 6%.

The limitation of the eighteen MR volumes of the IBSR database was that it had no Nasion point in the sagittal view. As we applied the proposed algorithm to retrospective MR volume with no Nasion point, the proposed algorithm could not test *X*-direction’s rotational effect.

### Evaluation of Brainweb Simulated Brain Database

We tested the proposed algorithm on a simulated dataset obtained from the BrainWeb Simulated Brain Database [26]. The simulated dataset consists of one T1-weighted MR images (slice thickness = 1 mm) each for normal and multiple sclerosis and T2-weighted MR images (slice thickness = 1 mm) of a normal brain.

**Fig. 8:**
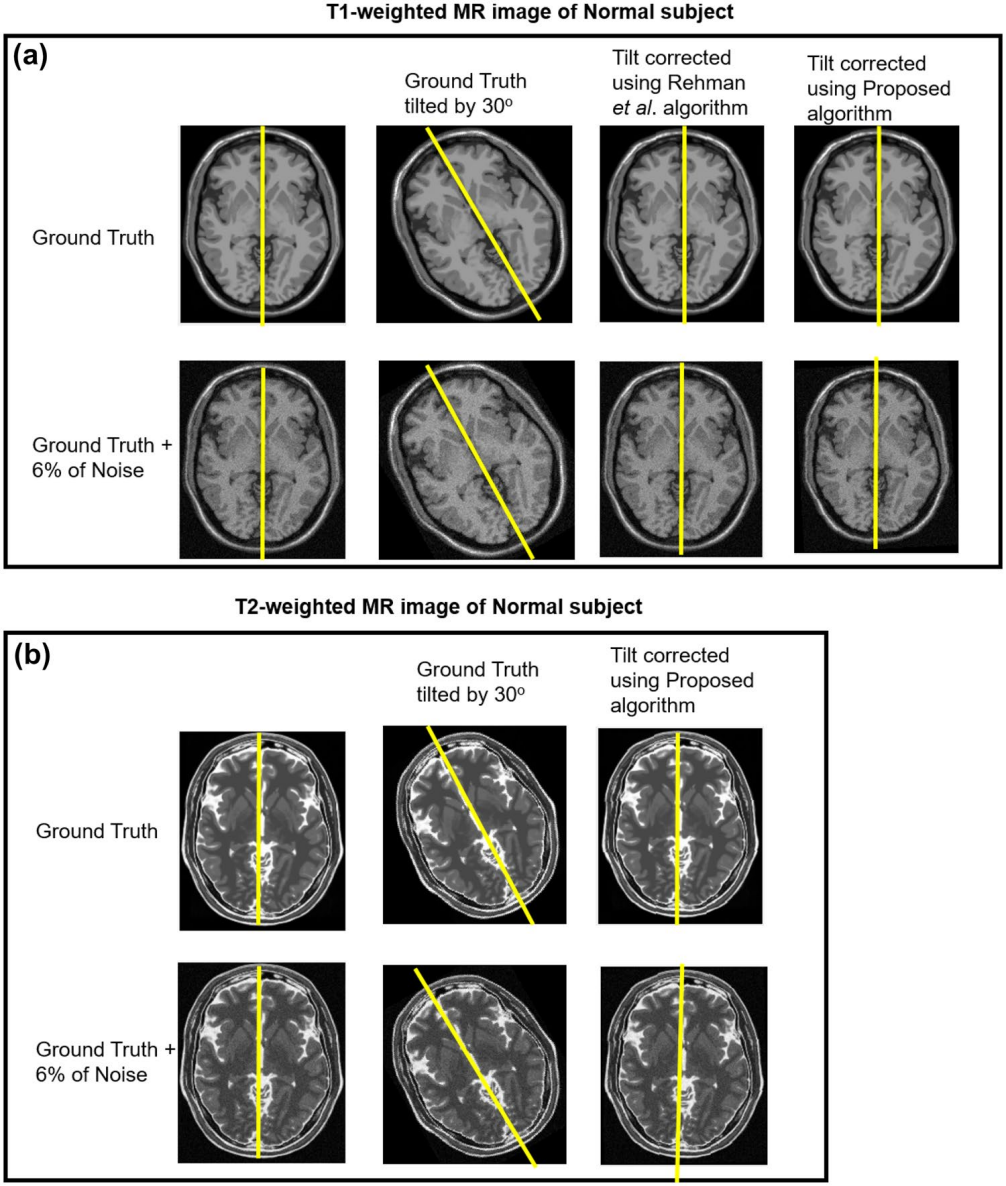
Evaluating the proposed algorithm on BrainWeb dataset: **(a)** on T1-weighted MR image of a normal subject and **(b)** on T2-weighted MR image of a normal subject

The proposed algorithm is tested on MR volume to understand the effect of different noise levels and varying slice thickness, influencing the tilt correction. Firstly, the T1-weighted MR images associated with a normal brain is rotated by 30° in *Y*-direction and *Z*-direction. The rotated T1-weighted MR image (of slice thickness = 1 mm) is corrupted with variation of 1%, 3%, 5%, 6%, 7%, 8%, and 9% of noise. The result showed that the proposed algorithm could successfully correct the tilt at below 6% noise level. Secondly, the T1-weighted MR image with the slice thickness of 3 mm, 5 mm, 7 mm, and 9 mm is rotated by 30° in *Y*-direction and *Z*-direction. We tested the proposed algorithm and algorithm by Rehman et al. [24] on the rotated T1-weighted MR image. The results showed that both algorithms failed to correct the tilt when the slice thickness is above 7 mm. The reason is that the brain contour does not exhibit an elliptical shape, due to which PCA fails to estimate the yaw angle, affecting the tilt correction. Overall, the proposed algorithm successfully corrects the tilt when the noise level is below 6%, and the slice thickness is below 7 mm. Figure 8 a demonstrates the tilt correction results performed on T1-weighted MR images for the noise level of 6% and slice thickness = 1 mm. Lastly, we tested the proposed algorithm and algorithm by Rehman et al*.* [24] for T1-weighted MR volume (0% noise and slice thickness = 1 mm) rotated by 30°, 20°, 10°, − 10°, − 20°, and − 30°. The algorithm by Rehman et al. [24] resulted in an error of 0.7° ± 0.3° after the tilt correction. The proposed algorithm corrected the tilt of simulated brain MR images with an error of 0.40° ± 0.03°. The error reported is the averaged error calculated for each angle of rotation.

The proposed algorithm was applied to the tilted T2- weighted MR image by 30° in *Z*- and *Y*-directions. The proposed approach corrected the tilt of MR volume with a variation of 0.5°. Figure 8 b shows the results of applying the proposed algorithm on T2-weighted MR images. When T2-weighted MR image (slice thickness = 1 mm) was corrupted with a noise level of 1%, 3%, 5%, 6%, 7%, 8%, and 9%. The proposed algorithm was successful in correcting the tilt with the noise level below 6%. We did not perform tilt correction about *X*-direction because the retrospective MR volume had no Nasion point. The proposed algorithm accurately and robustly corrects the tilt in *Y*-direction and *Z*-direction for a whole head MR volume.

### Evaluation of NIMHANS Dataset

**Fig. 9:**
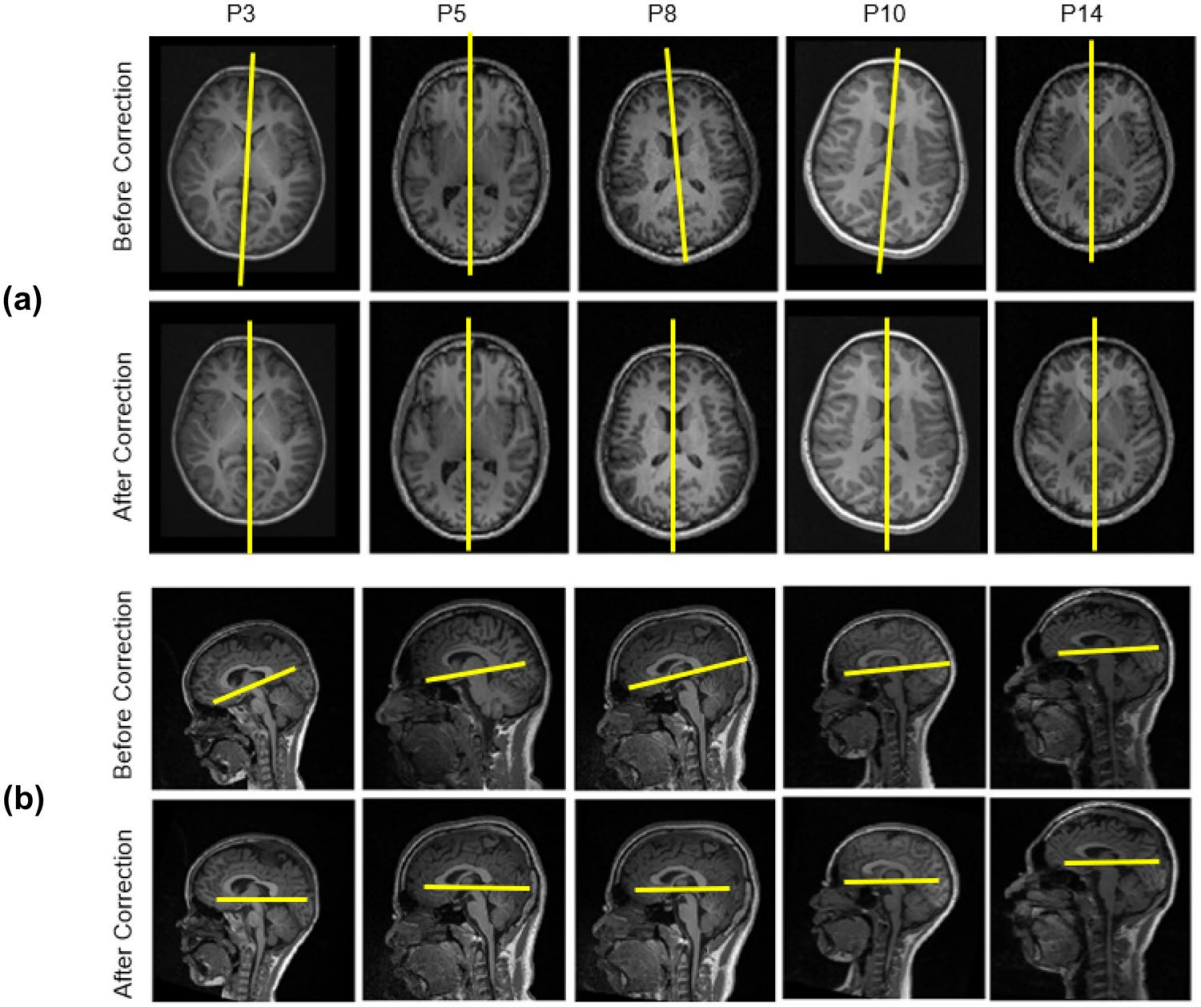
Realignment of brain MR volume using the proposed algorithm: (**a**) Realignment of MR volume of the five patients realigned in *Z*-direction and *Y*-direction. Visually inspecting the interhemispheric fissure in after and before correction helps to know the corrected tilt in *Z*-direction. Visually inspecting the symmetry helps to understand the corrected tilt in *Y*-direction and (**b**) the realignment of MR volume of the five subjects in *X*-direction. Visually inspecting the AC-PC line before and after correction helps to know the corrected tilt

We performed tilt correction on fourteen epilepsy patients’ T1-weighted images of MR volume using the proposed algorithm. Figure 9 a shows the correction performed in *Z*-direction and *Y*-direction.

Visual inspection of the interhemispheric fissure before and after correction helps to understand the correction performed in *Z*-direction and *Y*-direction. Figure 9 b shows the correction performed in *X*-direction. Visual inspection of the AC-PC line before and after correction helps to understand the correction performed in *X*-direction. The resulted pitch angle ranged from 6.2° ± 3.94°, the yaw angle ranged from 2.35° ± 2.61°, and the roll angle ranged from 5° ± 4.36° (as tabulated in Table 1). From the results, we can say that rotation about *Z*-direction experiences the rotation in both directions (negative and positive). The rotation about *Y*-direction has a considerable variation, which means that the patient inside the MR scanner shows sideways movement.

**Table 1:** Estimated pitch angle, yaw angle, and roll angle

Patient number	Yaw angle (degree)	Pitch angle (degree)	Roll angle (degree)
P1	3	9	17
P2	3	5	2
P3	6	15	2
P4	2	8	4
P5	0	4	12
P6	5	5	5
P7	6	2	3
P8	-2	8	5
P9	2	2	4
P10	3	12	0
P11	-2	7	3
P12	5	4	5
P13	1	5	4
P14	1	1	4

### Comparison of the Proposed Study with the Existing Studies

Ruppert et al. [21] used image processing techniques like Sobel edge detector and thresholding, followed by correlation to estimate the tilt about *Z*-direction (yaw angle). Rehman et al. [24] used PCA and similarity-based methods to estimate the tilt about *Z*-direction, and orthogonal regression to estimate tilt about *Y*-direction. Both the studies estimated tilt on T1-weighted MR images. The proposed study estimates the tilt in all three directions (*X*, *Y*, *Z*) in both T1- and T2-weighted MR images. The proposed work estimated the tilt in *X*-direction from the angle made by the line joining Nasion point to Inion point. Moreover, the proposed study automatically selects the axial slices that satisfy the properties of the ellipse. Whereas, in Rehman et al. [24], the middle few axial slices are manually selected based on the assumption that the head exhibit elliptical shape in these slices. Rehman et al. [24] study estimate the yaw angle on one axial slice by assuming that all the slices experience the same tilt. In reality, the subject inside the MR scanner might move the head, which leads to each slice undergoing different rotation. Therefore, the proposed study estimated the tilt in all the axial slices with an elliptical shape, and the averaged tilt angle was considered the final tilt about *Z*-direction. The proposed algorithm was successful in correcting the tilt not only in T1-weighted MR images but also in T2-weighted MR images for both normal and pathological conditions.

This study estimates the tilt in *X*-direction using the Nasion and Inion point; hence, the proposed algorithm demands the whole head MR volume. Some of the databases concentrate on the brain’s MR images, excluding the facial features; under such scenarios, the proposed algorithm is not suitable. These could be the limitation of this study. The algorithm by Ruppert et al. [21] is most likely not suitable, when the T1-weighted MR image experience tilt of about 30°, − 30°. Whereas, the algorithm by Rehman et al. [24] and the proposed algorithm can correct the tilt in the range [30°, − 30°], but the proposed algorithm is more accurate with an error less than a degree (error = 0.43° ± 0.09°). All three algorithms can tolerate up to a 6% level of noise to successfully correct the tilt about *Z*-direction and *Y*-direction. The algorithm by Rehman et al. [24] and the proposed algorithm can successfully correct the tilt for the MR images with a slice thickness of up to 7 mm. In addition to these, the proposed algorithm can correct the tilt in T2-weighted MR images and also MR images with pathological conditions.

### Advantages of using Mathematical Alignment to Correct the Tilt

For a successful MRI scan, the MR images acquired must be in the correct orientation. In other words, the axial centering of the MR volume is necessary. To achieve the correct orientation, the patient inside the gantry must remain still for the period of scanning. Certain patients like children, adults under medication, or in pain may not stay still during scanning under some circumstances. In such cases, the mathematical alignment of MR volume, as described in the proposed work, can achieve the correct orientation.

To obtain the correct orientation, it is not advisable to physically adjust the machine. This is because of two reasons. First, physically adjusting the machine is not possible because of the coils fixed within the machine. The distorted images result due to the movement of the coils. Secondly, the machine’s physical adjustment has to be done by technicians who need to know human anatomy, but that may not be the case in a real scenario. Meanwhile, physically adjusting the machine manually introduces subjectivity. Instead, the mathematical alignment described in the proposed study neither introduces subjectivity nor the images get distorted.

Currently, some of the third-party vendors are developing techniques to solve the problem of tilt correction. Siemens Healthineers is using the camera installed within the bore of MRI. This camera has the Kinetic sensors, which can detect the patient’s real-time motion within the gantry. Philips is trying to solve the problem of tilt correction using the deep learning model. They are using the 20,000 templates of the head to address the issue. Both the techniques offered by the imaging device vendors are under the development stage. Thus, there is no in-detail regarding the underlying techniques. The proposed approach is advantageous over the developing software to correct the tilt in retrospective data wherein the patient is no more available in the gantry to correct the tilt.

## Conclusion

MR volume suffers from tilt when a subject may not correctly align inside the gantry. The proposed study estimates the tilt in *Z*-direction, *Y*-direction, and *X*-direction with the aid of PCA, similarity-based measures, and image processing techniques. This study demonstrated the successful attempt to estimate the tilt with minimal error. The proposed work accurately and robustly corrects the tilt in the scenarios such as T1-weighted and T2-weighted MR image, MR image with the pathological case, and the simulated MR images. The proposed technique can automatically correct the tilt without any manual intervention, and the algorithm is computationally efficient. In general, the extended version of the proposed idea is to correct the tilt in the images used for multimodality registration.
